# Measuring Coverage in MNCH: Current Indicators for Measuring Coverage of Diarrhea Treatment Interventions and Opportunities for Improvement

**DOI:** 10.1371/journal.pmed.1001385

**Published:** 2013-05-07

**Authors:** Christa L. Fischer Walker, Olivier Fontaine, Robert E. Black

**Affiliations:** 1 Johns Hopkins Bloomberg School of Public Health, Department of International Health, Baltimore, Maryland, United States of America; 2 World Health Organization, Geneva, Switzerland (Retired); Wellcome Trust Senior Research Fellow in Clinical Science, UCL Reader in International Child Health, Honorary Consultant, Great Ormond Street Hospital for Children, United Kingdom

## Abstract

Diarrhea morbidity and mortality remain important child health problems in low- and middle-income countries. The treatment of diarrhea and accurate measurement of treatment coverage are critical if child mortality is going to continue to decline. In this review, we examine diarrhea treatment coverage indicators collected in two large-scale community-based household surveys—the Demographic and Health Surveys (DHS) and Multiple Indicator Cluster Surveys (MICS). Current surveys do not distinguish between children with mild diarrhea episodes and those at risk for dehydration. Additional disease severity questions may improve the identification of cases of severe diarrhea but research is needed to identify indicators with the highest sensitivity and specificity. We also review the current treatment indicators in these surveys and highlight three areas for improvement and research. First, specific questions on fluids other than oral rehydration salts (ORS) should be eliminated to refocus the treatment of dehydration on ORS and to prevent confusion between prevention and treatment of dehydration. Second, consistency across surveys and throughout translations is needed for questions about the caregiver behavior of “offering" the sick child fluid and food. Third, breastfeeding should be separated from other fluid and food questions to capture the frequency and duration of nursing sessions offered during the illness. Research is also needed to assess the accuracy of the current zinc indicator to determine if caregivers are correctly recalling zinc treatment for current and recent diarrhea episodes.

## Abstract 2

In a *PLOS Medicine* Review, Christa Fischer Walker and colleagues examine the diarrhea severity and treatment indicators that are currently collected in two large-scale community-based household surveys and suggest several ways to improve these coverage indicators.

*This paper is part of the* PLOS Medicine *“Measuring Coverage in MNCH" Collection*

## Introduction

Child diarrhea mortality rates have declined dramatically over the past 30 years, yet diarrhea morbidity has remained relatively constant among children under 5 years of age in low- and middle-income countries [1],[2]. Current estimates suggest that, despite improvements in water, sanitation, and hygiene, children under 5 years old have 2.9 episodes of diarrhea every year with the highest rates among children aged 6–11 months [2]. Rotavirus is widely accepted as the leading cause of hospitalizations among children under 5 years but other viruses, bacteria, and parasites also cause serious diarrheal morbidity and mortality [3],[4].

Oral rehydration salts (ORS) have been the cornerstone of diarrhea treatment since the 1980s [5]. Combined with continued feeding and the provision of home-based sugar-salt solution and other fluids, diarrhea treatment in the home should now be easier than ever for most community-acquired acute diarrhea episodes and experts had high hopes for accelerated uptake and widespread use of ORS within the community [6]. Unfortunately, although knowledge of ORS has remained high, more than two-thirds of low- and middle-income countries have reported declines in ORS use rates in the years following the initial campaigns and promotional efforts [7]. More positively, with the introduction of zinc supplementation for 10–14 days as an adjunct treatment for all episodes of childhood diarrhea [8], diarrhea treatment is now more effective than ever, and remains simple, inexpensive, and appropriate for community-based care.

A comprehensive understanding of which children with diarrhea are getting treatment and how this has or will change over time is critical for targeting child health programs. Our current understanding of the coverage of ORS and now zinc treatment comes primarily from Demographic and Health Surveys (DHS) and UNICEF Multiple Indicator Cluster Surveys (MICS). In this paper, which is part of the *PLOS Medicine* “Measuring Coverage in MNCH" Collection, we review the current methodology for assessing coverage of ORS, additional fluids, continued feeding, and zinc treatment. We identify problems with the currently accepted indicators and propose opportunities for improving these indicators. Better coverage indicators will increase our understanding of current trends in the treatment of diarrhea and enhance our ability to target and improve these interventions among young children.

## Overview of Current Methods for Assessing Coverage

DHS and MICS surveys have been measuring the coverage of key child survival interventions since 1984 and 1995, respectively [9]–[11]. These large, representative, cross-sectional household surveys are designed to track demographic and health indicators and to measure the coverage of key interventions in low- and middle-income countries. Details of the DHS and MICS survey methodologies are provided elsewhere in this Collection [11].

Except for the DHS in Peru and Senegal, which now employ continuous rolling surveys, DHS or MICS surveys are not executed in the field over a full calendar year. Because diarrhea disease rates are highly dependent on season [4], both surveys suffer from seasonality bias and cannot be used to accurately measure diarrhea incidence among children under 5 years of age [12],[13]. Cross-sectional surveys can, however, determine the standard denominator for measuring the coverage of key diarrhea treatment interventions—2-week diarrhea point prevalence. Thus, in DHS and MICS surveys, the caregivers of children under the age of 5 are asked if the child has had diarrhea at any time in the past 2 weeks. The questionnaire is designed to capture all episodes of diarrhea of varying degrees of severity, with or without blood in the stool, using local terminology for diarrhea rather than clinical definitions of disease duration or severity. Both DHS and MICS survey guidelines state that local terms should be included to capture the full spectrum of diarrhea as perceived in the local community.

DHS and MICS surveys also capture information on the coverage of key interventions for the treatment of diarrhea, namely ORS for the prevention and treatment of dehydration, zinc supplementation, continued feeding, and the provision of additional fluids during the episode. These treatment indicators are captured for all children with a diarrhea episode in the past 2 weeks. The caregiver is also asked about the quantity of fluids and foods that have been given during the current diarrhea episode in relation to what the child is normally given. The definitions of treatment coverage indicators most commonly used for program purposes are defined in Box 1.

**Box 1. Treatment Coverage Indicators Included in DHS and MICS Surveys**

*Indicators from direct responses to DHS/MICS questionnaires*

1. Proportion of children with diarrhea in last 2 weeks who were given ORS
2. Proportion of children with diarrhea in last 2 weeks who were given zinc
3. Proportion of children with diarrhea in last 2 weeks who were given recommended home fluids
4. Proportion of children with diarrhea in last 2 weeks who were given the same or more to drink
5. Proportion of children with diarrhea in last 2 weeks who were given the same or more to eat

*Indicators calculated from multiple DHS/MICS questions*

1. Proportion of children who received oral rehydration therapy (defined as ORS or recommended home fluids)
2. Proportion of children who received continued feeding and oral rehydration therapy or increased fluids

## The Definition of Diarrhea Episodes in DHS and MICS Surveys

The current denominator for treatment coverage obtained from DHS and MICS surveys includes all children with a diarrhea episode in the last 2 weeks as defined by the caregiver. Unfortunately, although considerable formative research was done in the early 1980s to understand local terms and beliefs about diarrhea [14], this information is often overlooked in the design of questionnaires and simple mistakes in the translation of survey instruments or the omission of key local terminology can result in missed diarrhea episodes. Moreover, current surveys assume that all diarrhea episodes are in need of the same level of treatment. Older surveys asked caregivers about the duration of the diarrhea episode, but this is no longer common. Currently, caregivers are asked about the presence of blood in the stool, but are not asked questions designed to classify disease severity, because this is thought to be difficult in household surveys with 2-week recall. Because the denominator broadly captures all diarrhea episodes, it is impossible to determine whether the children receiving appropriate treatment are the children most in need or simply a random sample of all children with diarrhea.

## How Can the Definition of Diarrhea Episodes Be Improved?

It is clear from the above discussion that large surveys need to include additional questions on diarrhea duration and severity to enable accurate measurement of treatment coverage. Such additional questions could use easily recognizable signs and symptoms, such as fever or abnormal thirst and vomiting to measure the prevalence of dehydration in the current or recalled episodes.

We identified two studies that showed various combinations of these signs as being predictive of dehydration [15],[16]. In a case control study in Brazil, researchers enrolled children who were hospitalized for dehydration as cases and age-matched children who also had diarrhea but were not hospitalized as controls. Mothers were asked to recount signs and symptoms on the first day of illness. The study concluded that using vomiting or fever as an indicator would identify 75% of diarrhea episodes with dehydration [15]. In a similar study in Mozambique, vomiting or fever had a sensitivity of 68.3% (sensitivity measures how well an indicator is able to identify true positive cases, i.e., diarrhea with dehydration). By adding “drinking more than usual" to this combination, the sensitivity of diarrhea prediction increased to 87.8% with a specificity of 34.1% (specificity measures how well an indicator is able to identify true negative cases, i.e., diarrhea without dehydration) [16]. We also found several studies that published risk factors for severe disease including socioeconomic factors, child characteristics, and some clinical signs and symptoms [17]–[19]. Although additional research is clearly needed to refine and retest these signs and symptoms before introducing new questions in large-scale surveys across low- and middle-income countries, these data suggest that qualifying diarrhea severity at the community level is possible.

With the objective of demonstrating that the addition of simple questions to large-scale surveys might provide valuable insights into diarrhea severity and into treatment by diarrhea severity, we used the sensitivities and specificities provided by Victora et al. [15] for combinations of reported signs and symptoms and 2×2 tables to calculate the positive and negative predictive values of these symptoms as predictors of dehydration during diarrhea. The positive predictive value (PPV) of a test indicates the proportion of individuals with a positive test result (here, diarrhea with dehydration) who actually have the disease being tested for. The negative predictive value (NPV) indicates the proportion of individuals with a negative test result who do not have the disease being tested for.

Victora et al. reported that the combination of “vomiting or fever or abnormal thirst" had the highest sensitivity (90%) and the lowest specificity (38%) (Table 1) [15]. The combination of “fever or vomiting" had the lowest reported sensitivity of 75% and the highest specificity of 66%. Typically, with any test, as sensitivity increases, specificity decreases and vice versa. Thus, as the signs or symptoms used to define diarrhea with dehydration become broader, more children meet the criteria of a “case" and the definition will capture a higher percentage of true cases. However, many more children who are not truly cases will also meet the case definition, which will increase false positivity and lower specificity.

**Table 1:** Range of diagnostic values for dehydration from diarrhea based on selected severity indicators.

Indicators	Sensitivity^a^	Specificity^a^	5% Prevalence of Dehydration		10% Prevalence of Dehydration	
			PPV	NPV	PPV	NPV
Thirst or fever or vomiting	90%	38%	7%	99%	14%	97%
Thirst or fever	89%	44%	8%	99%	15%	97%
Thirst or vomiting	89%	40%	7%	99%	14%	97%
Fever or vomiting	75%	66%	10%	98%	20%	96%

a Assumed sensitivity and specificity values are taken from Victora et al. (15).

Unlike sensitivity and specificity, PPV increases as the disease prevalence increases. We therefore tested scenarios with a 5% and a 10% prevalence of dehydrating diarrhea. At these relatively low prevalences, the PPV for the different combinations of signs and symptoms did not vary widely (Table 1). Thus, for this set of specificities and sensitivities, additional questions about the presence of vomiting, fever, and abnormal thirst could correctly identify 75%–90% (i.e., sensitivity can be high) of diarrhea cases most in need of ORS (i.e., diarrhea with dehydration), but of those individuals that appear to have severe diarrhea using these signs and symptoms as a set of indicators, less than 20% will truly have an episode of diarrhea with dehydration (i.e., PPV is low).

Additional validation studies are needed to test these and other possible indicators in several settings before any disease severity questions are universally added to surveys. The specific wording of questions will also need to be studied across several locations and in several diverse cultures. Although other risk factors for diarrhea or severe diarrhea have been identified [17]–[19], we suggest that the focus in DHS and MICS questionnaires should remain on signs and symptoms of the episode that are simple to identify and recall. In addition to those reported by Victora et al. [15], questions on total days with diarrhea for completed episodes, number of days of illness for current episodes, and stools per day may further define the severity of the diarrhea episodes and improve our understanding of differences, if any, with regard to treatment or care seeking. These questions should be evaluated to determine if adding them to the survey will increase specificity and sensitivity of identifying cases of diarrhea at risk of progressing to dehydration.

## Two-Week Point Prevalence of Diarrhea or Less?

Surveys are currently designed to capture a 2-week point prevalence of diarrhea, which assumes that recall of up to 2 weeks accurately captures both current and past episodes. Research has shown that longer recall periods actually underestimate milder diarrhea cases by approximately 40% and more severe cases by approximately 20% [20]. A shorter recall period may therefore be critical to more accurately describe diarrhea severity and duration even though a 1-week versus a 2-week recall would reduce the number of cases available for calculating treatment and care-seeking behaviors [21]. As with the inclusion of additional indicators to define severity, shifting the recall period in surveys from 2 weeks to 1 week needs to be tested before widespread changes are made. Making such changes may be logistically challenging given that DHS/MICS do not base sample size on diarrhea prevalence. Nevertheless, understanding the ideal recall period may still provide valuable information with regard to the coverage of diarrhea treatment interventions.

## Improving the Measurement of Coverage of ORS and Additional Fluids

Our review of the treatment indicators included in current DHS and MICS surveys revealed three major problems with the measurement of coverage of treatment with ORS and other fluids. Here, we describe these problems and recommend how they can be avoided in future surveys.

### Recommendation 1. Future Surveys Should Refocus on ORS

ORS is currently recommended for the *treatment* of all episodes of diarrhea. For cases of acute diarrhea with no signs of dehydration, fluids other than ORS may be used for the *prevention* of dehydration. The provision of additional fluids (except ORS) during a diarrhea episode was never intended as treatment for dehydration but, over time, the appropriate use of non-specific fluids in the management of dehydration has led to some confusion, and has also been poorly studied in general. For example, after the discovery of ORS, researchers tried to recreate a version of this life-saving intervention that could be made in the home if pre-packaged ORS was not available. Sugar-salt solutions were tested in hospitals against the packaged ORS formula and proved to be beneficial in clinical settings [22]. By contrast, evaluations of sugar-salt solutions in community settings were typically undertaken without control groups or comparison areas [23],[24]. Many other types of fluids have also been introduced into the category of home fluids without evidence to suggest a benefit [22].

Current surveys ask about ORS, “recommended home fluids," and the quantity of additional fluids, which includes plain water. However, the adaptation and translation of survey instruments for local use rarely inserts a description of locally recommended fluids containing a salt and starch into questions about “recommended home fluids." Instead, many surveys are implemented in the community with a direct translation of the phrase “recommended home fluids," which may generate meaningless responses. Furthermore, asking the caregiver about giving plain water and other fluids in separate questions does not provide information about the management of dehydration in the child.

In particular, the inclusion of the broad category of oral rehydration therapy, which includes feeding practices and the provision of nearly any liquid, as a valid indicator in surveys for the appropriate coverage of diarrhea treatment is questionable. The definition of this indicator has changed over time so looking at “oral rehydration therapy" in isolation of the definition describing the indicator does not allow accurate time trends to be described [25]. Moreover, with limited evidence suggesting a measured benefit of fluids other than ORS on diarrhea mortality, putting weight on this and other nebulous categories (for example, recommended home fluids) does not further our understanding of correct diarrhea treatment and will not do so until improvements in the denominator can help us differentiate which episodes of diarrhea can be managed with simple fluids and which episodes are in need of ORS for dehydration prevention and treatment.

We recommend, therefore, that questions about specific fluids other than ORS should be eliminated from large-scale household surveys. This will help to refocus attention on the importance of ORS and prevent confusion between appropriate treatments for preventing and treating dehydration.

### Recommendation 2. Future Surveys Should Focus on the Behavior of “Offering" the Child Fluid and Food

In large surveys the caregiver is asked if the child was offered or given ORS, additional fluids, or food. It is often difficult to distinguish which question—was the child “given" (i.e., consumed) versus was the child “offered"—has been asked in the translated version(s) of the questionnaire, even though back translation is routinely undertaken to check the accuracy of translations. Indeed, in some languages, there might not even be words that adequately distinguish between these two concepts. Moreover, reports of surveys may not accurately recount what was asked of the caregiver or, in some instances, may use both terms [26]. We suggest that the objective of the question in large surveys should be whether or not the child was offered ORS, additional fluids, or food. This is the behavior that is in the control of the caregiver—sick children may not take all that is offered. Smaller-scale surveys may be able to ask about the intake of the child to further our understanding of caregiver behaviors that may influence the child's response to food and drink being offered during the illness, but large DHS and MICS surveys will not be able to include both sets of questions.

In many countries, it is still the cultural norm to restrict feeding during the diarrhea episode or to change the food offered to those that are perceived as easy to digest. It is often thought that “less in" will mean “less out." Current recommendations emphasize the importance of continued feeding during the diarrhea episode [8] to ensure the child receives adequate nutrition. Secondary questions that ask caregivers if the child was offered/given the same, more, or less fluids and food than usual are already included in DHS and MICS surveys but again, it is important to ensure that these questions are consistently translated to ensure that the data gathered accurately reflects the concept of food and fluid offered and does not get confused with quantities eaten or drank. Quantifying exact quantities of food or fluid ingested is difficult to ascertain correctly from a household survey; therefore the concept of offered fluids and foods remains the best indicator of progress for programmatic consideration [27],[28]. Furthermore, although a better understanding of changes in feeding practices with regard to what foods a child is offered during diarrhea is needed to ensure that appropriate nutrition is maintained during the episode, particularly in children who have longer episodes or multiple episodes, we believe it is inappropriate to include any other questions related to feeding practices in large surveys; instead smaller research studies should study feeding practices during diarrhea episodes to identify populations most at risk of malnutrition because of diarrhea.

### Recommendation 3. Future Surveys Should Separate Breastfeeding from Other Fluids and Foods

Current questionnaires include breast milk as part of the question dealing with the quantity of fluids offered. However, the quantity of breast milk cannot be measured if the child is fed at the breast. A mother can only report frequency of nursing opportunities or time spent nursing and changes in nursing patterns. We suggest that a separate question that asks if the child was given opportunities to nurse the same, more, or less frequently and/or for more, the same, or less time during the illness would allow the mother to more accurately describe the child's nursing pattern in lieu of providing information on the quantity of breast milk ingested.

## Coverage of Zinc Supplementation in DHS and MICS Surveys

Although zinc supplementation was added to the UNICEF/WHO recommendation in 2004, it has still not been incorporated widely into diarrhea treatment programs [6],[8]. Indicators of zinc supplementation have been added to the routine DHS and MICS surveys but little testing has been done to determine whether caregivers can differentiate zinc from more commonly prescribed antibiotics and antidiarrheals in communities where zinc is a new treatment, particularly when recalling an episode in the past 2 weeks that has resolved and for which treatment is no longer being given. Caregivers may be able to more accurately recall treatments given if the recall period is shortened. A shorter recall period would result in a greater number of children currently receiving treatment and thus enable the surveyor to ask to see the packaging of the treatment being given to validate the caregiver's response. On the downside, a shorter recall period would limit the sample size and prevent opportunities for stratified analyses. As mentioned earlier in the context of reducing the recall period to improve reporting of diarrhea severity, research is needed to test whether shorter recall periods improve the accuracy of caregiver reports of zinc supplementation. Notably, few studies to date have examined the appropriate recall time for treatments given for childhood diseases. Additional research is also needed to better understand other aspects of the ability of caregivers to correctly identify and recount giving zinc for the diarrhea treatment, especially during the early years of introducing zinc into routine clinical practice.

## Conclusions

Current diarrhea coverage indicators seek to capture the coverage of zinc treatment and fluid replacement for the prevention and treatment of dehydration. Because many of the current indicators have not changed over time, tracking coverage over time is possible. Nevertheless, in this paper, we present several opportunities for improving upon currently accepted indictors. We recognize that the scientific evidence supporting the methods for improvements we propose is lacking in most cases (Box 2). For this reason, this Review should be viewed as a call to action rather than as a guide to changes that should be made immediately.

**Box 2. Recommended Changes to Current Diarrhea Coverage Indicators**

*Recommended changes requiring no additional research*

1. Eliminate questions with regard to recommended home fluids and renew focus on ORS.
2. Ensure questions in DHS and MICS that focus on “offering" a child fluids/food are clearly differentiated from questions that concern fluid/food intake.
3. Separate breastfeeding from other fluids and foods in all questions.

*Recommended changes requiring field-testing or additional research*

1. Develop and test the validity of including selected diarrhea severity questions as part of large household surveys.
2. Determine if 1-week recall can accurately collect information with regard to diarrhea severity in addition to basic coverage data.
3. Determine if caregiver recall of zinc supplementation in both tablet and syrup forms is accurate in a 2-week recall questionnaire.

Our motivation for suggesting changes to coverage indicators is driven by a need to better understand who and what types of diarrhea are being treated appropriately and to identify the episodes of diarrhea most likely to progress to dehydration. Diarrhea remains an important cause of morbidity and mortality for children in low- and middle-income countries around the world, and diarrhea treatment rates do not appear to be improving. However, it is possible that the children who need ORS most are now receiving adequate treatment and that this has contributed in part to the recent decline in diarrhea mortality [1].

Enhanced coverage indicators should enable us to better track changes in coverage over time. Importantly, however, because DHS/MICS surveys are already long, additional questions should not be added without considerable reflection on the cost-benefit of the new data collected. The ability of the changes proposed here to improve the measurement of treatment coverage for diarrhea must also be carefully studied before inclusion in routine DHS/MICS surveys to ensure that these surveys remain valuable tools without becoming too burdensome for practical use.

Finally, we acknowledge that making the changes we propose will inevitably mean that new indicators will not be comparable to past indicators. We suggest that this limitation may be a reasonable price to pay for improvements in our understanding of how well programs are targeting the children and the diarrhea episodes most in need of appropriate treatment.

**Key Points**

- Diarrhea morbidity and mortality remain important child health problems in low- and middle-income countries.
- Additional research is needed to determine the most appropriate signs and symptoms to include in household surveys to enable the distinction between mild and more severe diarrhea to be made.
- Simplification of current surveys to renew/increase the focus on oral rehydration salts, on the behavior of offering a child fluids and food throughout their illness, and on breastfeeding should improve the measurement of treatment coverage.
- Zinc for the treatment of diarrhea is new and research is needed to understand the ability of caregivers to recognize and accurately recall zinc treatment for diarrhea
- By improving coverage indicators, we will increase our understanding of trends in diarrhea morbidity and mortality and be able to target interventions better.

## Abbreviations

DHS: Demographic and Health Survey/s
MICS: Multiple Indicator Cluster Survey/s
MNCH: maternal, newborn, and child health
NPV: negative predictive value
ORS: oral rehydration salts
PPV: positive predictive value
